# Accurate contextual reporting amidst dynamic AI curricula development: A response to Zainal and colleagues

**DOI:** 10.1111/medu.70241

**Published:** 2026-05-13

**Authors:** Li Yunkai Andrew, Quek Yong Jing Daniel, Minyang Chow, Bernett Lee, Jennifer Cleland, Chia Faith Li‐Ann

**Affiliations:** ^1^ Lee Kong Chian School of Medicine Nanyang Technological University Singapore

Dear Editor,

We read Zainal and colleague's article on a digital‐age clinical artificial intelligence (AI) ethics competence framework with interest.^1^ We agree with their central argument—AI ethics must be addressed explicitly and practice‐grounded in medical training. However, the paper's reporting of contextual information raises concerns of general importance.

Table 1 describes the context of AI and ethics integration in Singapore's three medical schools. Presumably, this context was collected prior to Zainal and colleague's data collection (April–June 2025).^1^ The AI curriculum at Lee Kong Chian School of Medicine (LKCMedicine) was described as ‘still in development’. Yet, LKCMedicine had integrated digital health and AI into its curriculum in 2020 and extended this as part of a major MBBS curriculum reform which included the explicit learning objective of students developing a ‘firm foundation in the ethical and legal consequences of AI and healthcare informatics’.^2^ This new course was publicly announced in 2023 and implemented in 2024.

This discrepancy foregrounds three general methodological issues.

First is the need for rigorous descriptions of context.^3^ Although Zainal and colleagues made clear that the information was acquired separately from the main qualitative data collection, we believe that it is critical to know details of the approach used in gathering such information, ranging from the consultation/data collection timing to the ‘information power’ of those consulted,^4^ to how information verification and contextualization was conducted. This is particularly important when explanations of self‐reported experience are interpreted against contextual information.

Second, any statement about curricular content can only capture the status quo at a single time point. In rapidly developing content domains such as AI in medicine/medical education which necessitate ongoing curricular evolution, such published descriptions, which are often perceived as ‘permanent’, may lead to misjudgments about the maturity and responsiveness of a curriculum, potentially causing credibility damage. For this reason, authors must make the timing of their information gathering explicit.

Third, studies of AI in health professions education must shift from snapshots in time and self‐reporting to examining and exploring the complexity of integrating AI‐related content into crowded traditional curriculum and the processes and impact of doing so.

Zainal and colleagues' paper is part of a body of research focused on preparing tomorrow's doctors for AI use in clinical practice. In parallel, we call for informed and ethical reporting of contextual information in AI and all health professions education studies which recognise the complexity of change.

## AUTHOR CONTRIBUTIONS

Andrew Li Yunkai is Assistant Professor at Lee Kong Chian School of Medicine and is co‐chairperson of the AI enhanced Education workgroup. Quek Yong Jing Daniel is Assistant Professor at Lee Kong Chian School of Medicine and is co‐chairperson of the AI enhanced Education workgroup. Minyang Chow is Assistant Professor at Lee Kong Chian School of Medicine. Bernett Lee is Assistant Professor of Biomedical Informatics and Director, Centre for Biomedical Informatics, Lee Kong Chian School of Medicine. Jennifer Cleland is Professor, Medical Education Research and Director of Medical Education Research & Scholarship Unit (MERSU) and the President's Chair in Medical Education at Lee Kong Chian School of Medicine. She is also the Editor‐In‐Chief of Medical Teacher.

Faith Chia Li‐Ann is Associate Professor and Vice Dean Education at Lee Kong Chian School of Medicine. Minyang Chow penned the first draft. All other authors contributed to the revision of this manuscript and provided supervision.

## CONFLICT OF INTEREST STATEMENT

All authors declare no competing interests.

## ETHICS STATEMENT

Not applicable. This article does not involve human or animal participants.

## GENERATIVE AI DISCLOSURE

Generative AI was used for editorial refinement. The authors retained full control and verified all content prior to submission.

## Data Availability

Data sharing is not applicable to this article as no datasets were generated or analysed during the current study.
